# CD5, an Undercover Regulator of TCR Signaling

**DOI:** 10.3389/fimmu.2018.02900

**Published:** 2018-12-07

**Authors:** Guillaume Voisinne, Anne Gonzalez de Peredo, Romain Roncagalli

**Affiliations:** ^1^Centre d'Immunologie de Marseille-Luminy, Aix Marseille Université, INSERM, CNRS, Marseille, France; ^2^Institut de Pharmacologie et de Biologie Structurale, Département Biologie Structural Biophysique, Protéomique Génopole Toulouse Midi Pyrénées CNRS UMR 5089, Toulouse, France

**Keywords:** CD5, TCR-T cell receptor, signaling/signaling pathways, coreceptor, inhibition

## Abstract

T cells are critical components of adaptive immunity. As such, their activation is regulated by the T cell receptor (TCR) that constantly scan peptides associated with major histocompatibility complexes (MHC). TCR engagement initiates a series of molecular events leading to cytokine secretion, proliferation, and differentiation of T cells. As a second coincident event, activation of co-stimulatory molecules, such as CD28, synergize with the TCR in order to prolong and/or amplify intracellular signals. With the recent advances in immunotherapies targeting T cells, co-inhibitory receptors are of growing interest for immunologists due to their potential modulatory properties on T cell functions. However, special attention should be dedicated to avoid unwanted clinical outcomes (1). In particular, Manichean categorization of receptors based on incomplete functional knowledge can lead to an over-simplistic view of complex cellular regulations. Thus, analysis of the functions that characterize these receptors in diverse physiological contexts remains essential for their rational use in therapeutic protocols. Here we focus on CD5, a transmembrane receptor that regulates T cell functions and development but remains poorly characterized at the molecular level. We will review its roles in physiological conditions and suggest potential molecular effectors that could account for CD5-dependent regulation of TCR signaling.

## Regulation of T Cell Development and Function by CD5

Seminal studies have identified CD5 as an activation marker of T cells (2, 3). Thus, expression of CD5 increases according to the magnitude of the signal delivered by the TCR. Consequently, CD5 expression reflects the heterogeneity of the signal strength associated with each individual TCR within a polyclonal T cell population. This observation has been also documented with various TCR transgenic mice, for which CD5 expression levels correlated with the affinity of the TCR with its known agonist peptide (4, 5). The study of CD5 deficient mice allowed to position CD5 not only as an activation marker but also as an active player of the TCR signaling pathway (6). Indeed, absence of CD5 enhanced signaling and activation of double positive (DP) thymocytes induced by TCR stimulation. Moreover, CD5 deficient DP thymocytes from TCR transgenic mice have a shifted windows of selection toward a lower threshold, resulting in an enhanced positive or negative selection with TCRs of low or high avidities, respectively (7, 8). These results established CD5 as a negative regulator of the TCR signaling pathway in immature thymocytes.

In contrast to its role in the thymus, the functions of CD5 in the periphery remain unclear. On the one hand CD5 deficient peripheral T cells showed better proliferative responses following TCR stimulation than their wild-type counterparts (8), suggesting that CD5 also acts as a negative regulator of TCR signaling in mature T cells. On the other hand, analysis of polyclonal and TCR transgenic T cells showed that effector functions of mature T cells positively correlate with CD5 expression (4, 5, 9). As a result, it has been proposed that the abundance of CD5 can predict TCR avidities with self and foreign peptides (4). Also, other reports suggested that CD5^hi^ cells acquired intrinsic properties during thymic selection against self-peptides that could be maintained in periphery leading to improved reactivity against foreign antigens (5, 9).

These complex results illustrate the difficulty of assessing the impact of altered thymic selection on T cell reactivity in the periphery. Indeed, comparing results obtained in the periphery and in the thymus raises several issues. The first issue is that phenotypic differences observed in peripheral T cells could result from perturbed thymocyte education. Hence, in the case of CD5 deficient mice, the increased proliferation observed in periphery could be due to an alteration of the TCR repertoire selected during thymic development. Also, changes in selection pressure could modify the abundance of other regulators of the TCR signaling pathway. For example, it has been shown that the abundance of CD6 (a transmembrane receptor structurally related to CD5) was higher in peripheral T cells deficient for CD5 (10).The second issue is related to the difficulty of tracking the same cell during the processes of thymic selection and egression *in vivo*. Thus, although CD5 expression correlates with the magnitude of the TCR signal during DP selection and of tonic TCR signals in periphery, it does not necessarily indicate that a CD5^hi^ cell in the thymus remains CD5^hi^ in the periphery. Indeed, it is possible that selecting self-peptides are absent in the periphery or do not induce a similar TCR reactivity as they did during thymocyte selection. The above issues make it difficult to distinguish between direct CD5 signaling effects in peripheral T cells from indirect consequences of perturbed thymic selection. Conditional deletion of CD5 in peripheral T cells would greatly help elucidate the role of CD5 in periphery independently of its effect on thymic selection.

## Structural Basis for the Negative Regulation Exerted by CD5

From a structural point of view, CD5 is a type-I transmembrane glycoprotein with an extracellular region composed of three scavenger receptor cysteine-rich (SRCR) domains. Several CD5 ligands have been reported such as CD72, the IgV(H) frame-work region and several polypeptides (gp40-80, gp150) whose identity remains undetermined (3, 11, 12). CD5 can also establish low stoichiometric homophilic interactions in *cis* or in *trans* (13). Whether these molecules bind to CD5 and modulate its activity in physiological settings remains a matter of debate. Even so, it has been reported that cross-linking of antibodies targeting the extracellular domain of CD5 induces signaling in the Jurkat cell line (14). In the absence of binding of CD5 with potential ligands, TCR stimulation triggers CD5 phosphorylation on tyrosine residues (15) and its translocation into the immunological synapse (16), thereby indicating a direct regulation of CD5 by TCR signals. Both types of stimulation suggest that CD5-mediated signaling inhibition could be potentiated by spatial confinement in areas where phosphatases are excluded and kinases enriched.

On its cytoplasmic tail, CD5 contains four tyrosine residues at position 402, 453, 464, and 486 in human (historically Y378, Y429, Y441, and Y463 if the signal peptide sequence is not included) exposed to potential phosphorylation regulations. Although the tyrosine Y402 was initially associated with the CD5 inhibitory signal through its association with the SH2 domain containing-tyrosine phosphatase 1 (SHP-1) (17), cumulative data from mass spectrometry analysis failed to detect phosphorylation at this position even though the corresponding peptide bearing this tyrosine residue is frequently observed (source: phosphosite.org and peptideatlas.org). In contrast, the three distal tyrosine residues (Y453, Y464, and Y486) have been frequently observed in their phosphorylated form. Moreover, studies using either phosphopeptides coding for CD5 tyrosine motifs or B cells transfected with a chimeric molecule composed of the extracellular and the transmembrane domains of FcgRIIB with the cytoplasmic domain of CD5 did not detect SHP-1 interaction (18, 19). Consistently, analysis of truncated mutants of CD5 demonstrated that the cytoplasmic tail of CD5 comprising these three distal tyrosines residues could account for global CD5 phosphorylation following pervanadate stimulation and was required for CD5 signaling activity (7). These three distal tyrosine residues are subjected to Src kinases regulation and have been proposed as docking sites for several effectors such as the RasGAP or the phosphatidylinositol 3-kinase (PI3K) (18, 20). In addition to the tyrosine dependent interactions, it has been shown that the two carboxy-terminal serine residues of CD5 allow constitutive binding with the casein kinase 2 (CK2) (21). Transgenic mouse models for which the CD5 serine motif has been deleted display abnormal T cell development and perturbed differentiation of mature T cells (22, 23). Moreover, T cells from these mice exhibit reduced survival capacity and hypoproliferate in response to TCR stimulation. These studies illustrate that CD5 signal transduction relies on both tyrosine and serine motifs.

More recently, our group demonstrated that CD5 could associate with CBL, CBLB, and GRB2 in mature CD4^+^ T cells upon TCR stimulation (24, 25). To do so, we developed mouse models suitable for proteomics analysis in primary T lymphocytes. These mice are genetically engineered to express proteins bearing an OST tag at their N terminal, thereby serving as “baits” allowing affinity purification (AP) of protein complexes. AP samples are subjected to tandem mass spectrometry (MS-MS) and specific binding partners are identified by comparing protein intensities in samples from cells bearing the endogenous or the OST-tagged proteins. Using this approach, the set of specific binding partners for a protein of interest, its “interactome,” can be quantified in a comprehensive manner. We discuss in the following how the molecular mechanisms of CD5 signaling might be revisited in light of these recent results.

## Cooperativity Between CD5 and the Ubiquitin Ligases CBL and CBLB in Mature T Cells

CBL molecules (CBL and CBLB) are E3-ubiquitin ligases involved in the negative regulation of the TCR signaling pathway via different complementary mechanisms (26). CBL has been shown to control ubiquitination and degradation of the CD3 chains and activities of the proximal tyrosine kinases LCK and ZAP70 (27–29) whereas CBLB negatively regulates the CD28 co-stimulatory pathway by dampening the PI3K activity (30–32).

CBL and CBLB both target specific substrates for ubiquitination. Globally, CBLB proximal molecular environment contains more ubiquitinated species than CBL, suggesting a predominant role of CBLB over CBL for this post-translational regulation in mature T cell (24). This observation correlated with the severe phenotype of the *Cbl-b*^−/−^ T cells exhibiting an increased capacity to proliferate and secrete cytokines when activated (30, 31).

**Figure 1:**
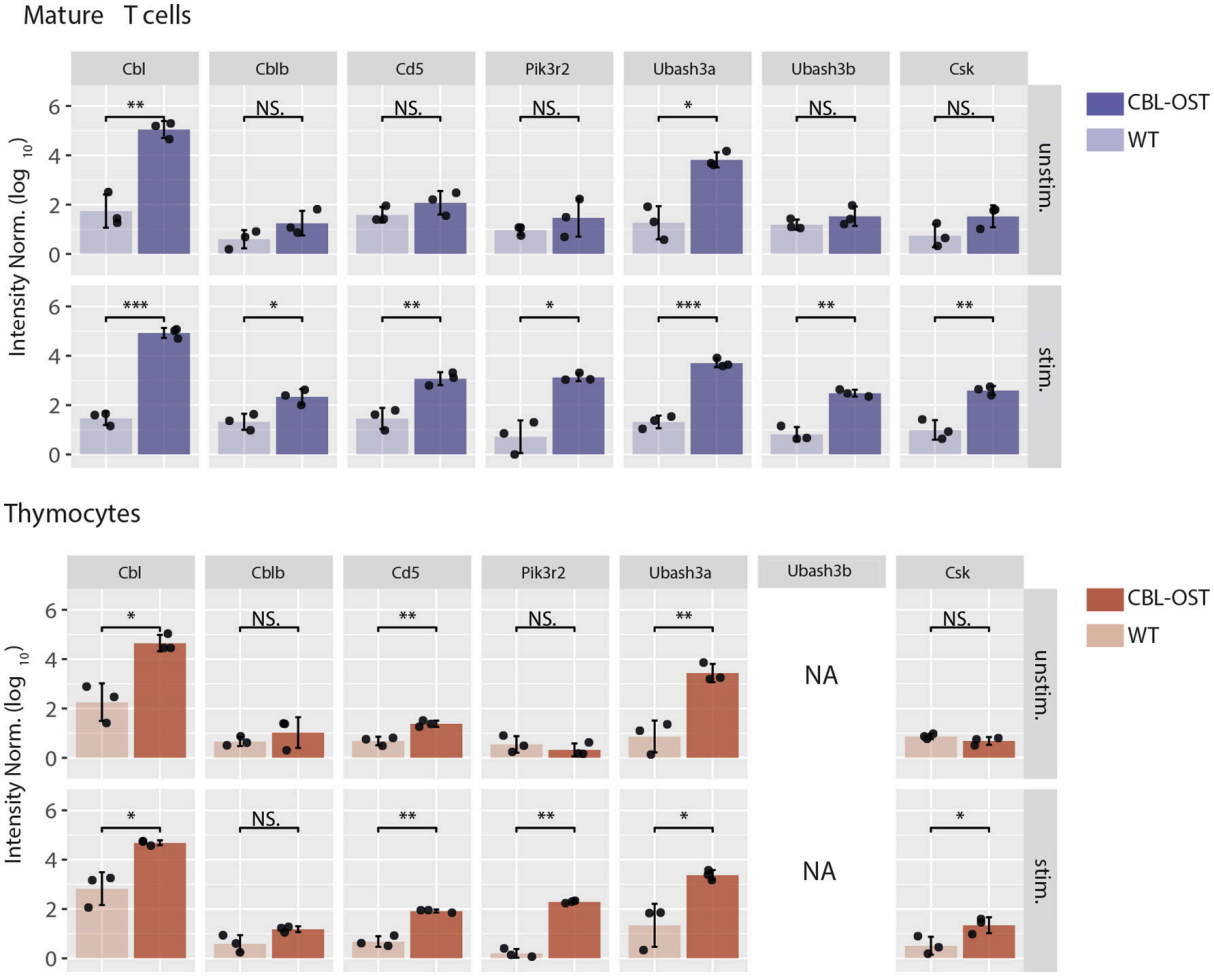
Association of selected proteins with CBL in thymocytes and mature T cells. Mature CD4^+^ T cells and thymocytes from wild-type (WT) and gene-targeted mice expressing One-STrEP-tag at the carboxyl-terminus of endogenous CBL (CBL-OST) were left unstimulated (unstim.) or stimulated for 30 s (stim.) with anti-CD3 plus anti-CD4 antibodies and subsequently lysed. Protein lysates were subjected to OST affinity purification coupled to mass spectrometry analysis (AP-MS) (24, 34). For each sample, protein intensities were log transformed and normalized by the sample median intensity. Intensities were then averaged across technical replicates and missing values imputed by values simulating noise around the detection limit. After missing values imputation, log-transformed intensities from WT and CBL-OST cells were compared using a two-sided Welch *t*-test (symbols used according to the *t*-test *P*-value: N.S., *P* > 0.05; *P ≤ 0.05; **P ≤ 0.01; ***P ≤ 0.001). Intensities were divided by the minimum intensity across all intensities represented to ensure that all log-transformed values were positive. Data used for mature CD4^+^ T cells are from Voisinne et al. (24) (NA, non-applicable).

Because CBL and CBLB interact together (24, 33) (Figure 1), it is possible that the scaffolding property of each ubiquitin ligase allows *trans*-ubiquitination of contiguous proteins not subjected to *cis*-ubiquitination. For example, although PI3K subunits are specifically associated with CBL in peripheral T cells, their ubiquitination is mainly regulated by CBLB (24, 32).

In peripheral T cells, both CBL and CBLB associated with CD5 upon TCR stimulation (24). This suggests that CD5 could play a scaffolding role, facilitating the CBL-CBLB relocalization to the plasma membrane in proximity of the tyrosine kinases required for their activities. This cooperativity between CBL, CBLB, and CD5 could also be important for enhancing ubiquitination within supra molecular complexes assembled upon TCR stimulation. In line with this model, mature T cell from CD5 deficient mouse showed reduced CBL-dependent ubiquitination in activated T cells (24). More specifically, ubiquitination of PI3K subunits following TCR stimulation was reduced in the absence of CD5 suggesting that CD5 could facilitate *trans*-ubiquitination.

In the CBLB deficient T cells, association of CD5 with CBL was preserved. Interestingly, the absence of CBLB enhanced the interaction between CD5 and CBL and increased global protein ubiquitination within the complex formed around CBL (24). These results suggest that CBL molecules compete for binding to shared docking sites on CD5 and in the ubiquitination of shared substrates. Also, they indicate that despite a molecular reorganization in the absence of CBLB, CBL is unable to fully compensate for CBLB deficiency in mature T cells. Hence, in peripheral T cells, CD5 could negatively control TCR signaling by coordinating ubiquitination through its interaction with CBL and CBLB.

## Interactions Between CD5 and CBL in Thymocytes

**Figure 2:**
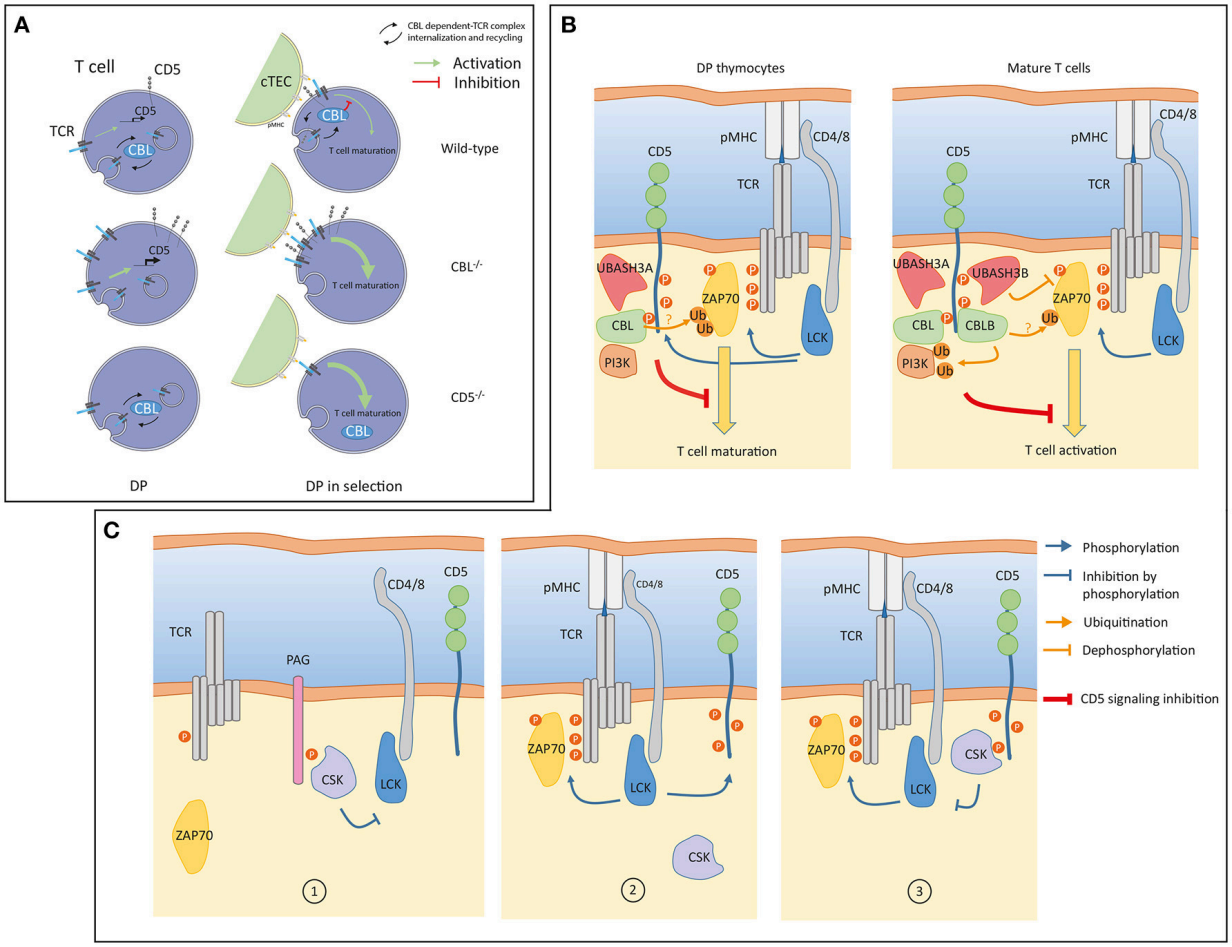
**(A)** A model of signaling in double positive (DP) thymocytes from wild-type, *CBL*^−/−^ and *CD5*^−/−^ mice. Prior selection (left), constitutive TCR internalization, recycling and degradation are regulated by CBL. CD5 is not involved in these processes but CD5 protein level is transcriptionally controlled by weak constitutive TCR signaling (green arrow). In the absence of CBL, surface TCR concentration increases which enhances transcription of CD5. Surface TCR concentration is unaffected in CD5 deficient cells and remains controlled by CBL. During selection (right), CBL associates with CD5 within the immunological synapse (IS) to negatively control TCR signaling. In the absence of CBL, TCRs accumulate at the cell surface leading to increased TCR signaling. The inhibition of TCR signaling by CD5 is impaired in the absence of CBL. In CD5 deficient cells, recruitment of CBL to the immunological synapse (IS) is impaired which leads to enhanced TCR signaling. (cTEC: Cortical thymic epithelial cell) **(B)** A model of CD5 signaling in thymocytes and mature T cells. Upon TCR engagement, LCK phosphorylates the CD3 chains and CD5 (blue arrows). Phosphorylation of CD5 allows interactions of inhibitory molecules such as CBL and UBASH3 proteins triggering post-translation modifications (ubiquitination, dephosphorylation) of positive effectors (ZAP70, PI3K) involved in the proximal TCR signaling pathway. The global negative signal mediated by CD5 is symbolized by the inhibitory red line. In thymocytes, CBLB and UBASH3B expressions are undetectable, CD5 associated only with CBL and UBASH3A. The PI3K interacts with CBL in thymocytes and in peripheral T cells. PI3K regulation by ubiquitination is essentially mediated by CBLB. **(C)** An alternative model of CD5 signaling involving CSK. In quiescent T cells CSK interacts with PAG to negatively control LCK (1). Upon TCR engagement LCK phosphorylates CD5 (2). CSK molecules associated with phosphorylated CD5 localized into the IS. CD5-associated CSK phosphorylates the inhibitory tyrosine residue of LCK thereby reducing the magnitude of TCR signaling (3). Panel **(A)** was modified from Servier Medical Art, licensed under a Creative Common Attribution 3.0 Generic License. http://smart.servier.com/.

The situation described above is modified in thymocytes where abundances of CBL molecules differ from that in mature T cells. While CBL and CBLB have similar abundances in peripheral T cells, protein expression of CBLB is much lower than CBL in thymocytes (24). In agreement with observations reported in CBLB deficient mature T cells, analysis of the CBL interactome in thymocytes revealed that the association between CD5 and CBL is maintained despite the low abundance of CBLB (Figure 1). These results suggest the existence of a functional relationship between CBL and CD5 in thymocytes. The comparison of the phenotypes between the CBL and CD5 deficient mice partially supports this hypothesis. Indeed, both CBL and CD5 deficient DP thymocytes show enhanced intracellular signaling which, onto a low avidity TCR transgenic background, lead to increased positive selection (35, 36). However, in contrast to *CD5*^−/−^ mice, DP thymocytes of *CBL*^−/−^ mice have elevated TCR levels due to reduced TCR degradation and increased TCR recycling (37, 38). Thus, increased TCR reactivity in *CBL*^−/−^ DP thymocytes could essentially reflects the increased abundance of the TCR at the plasma membrane. This phenotype might mask another function of CBL. Indeed, considering that the interaction between CBL and CD5 depends on TCR stimulation, the increased TCR responses observed in CD5 deficient mouse could reflect a specific role of CBL strictly dependent on TCR engagement. Hence, two mechanisms of TCR signaling regulation involving CBL could coexist (Figure 2A). One where CBL, independently of CD5, regulates the constitutive TCR pool at the surface of DP thymocytes and another one, triggered by the TCR stimulation, relocating a fraction of CBL molecules to the synapse via CD5 and promoting its inhibitory activity (ubiquitination) in this particular cellular localization. In this molecular context, specific effectors of the proximal TCR signaling pathway could be negatively controlled by CBL.

Hence, both in thymocytes and in mature T cells, CBL molecules are possible molecular mediators of CD5 inhibition of TCR signaling (Figure 2B).

## Contributions of UBASH3A/B Molecules to CD5 Inhibition

Other molecules than CD5 associated with both CBL and CBLB in TCR stimulated mature T cells (24). Among them, the Ubiquitin-associated and SH3 domain-containing protein A and B (UBASH3A, UBASH3B also known as STS-2 and STS-1) have been associated with negative regulation of the TCR signaling pathway (39) and might therefore participate in CD5 inhibition.

Association of CBL with UBASH3A was detected in unstimulated thymocytes and mature T cells and remained unchanged upon TCR stimulation (Figure 1). In contrast, UBASH3B was associated with CBL only upon TCR stimulation and this recruitment correlated with that of CBLB. This suggests preferential associations of CBL with UBASH3A and CBLB with UBASH3B. In support of this statement, the expression pattern of UBASH3A and UBASH3B proteins in alpha/beta T cells is similar to that of CBL and CBLB, respectively (39) (www.immgen.org). Hence, UBASH3A is highly expressed in DP thymocytes whereas expression of UBASH3B starts in single positive (SP) thymocytes.

Both UBASH3 molecules exert phosphatase activities and bind ubiquitinated proteins though their UBA domains (40, 41). When both molecules are inactivated, mature T cells showed an enhanced capacity to proliferate and secrete cytokines, a phenotype reminiscent of those observed with CD5 and CBLB deficient mice (39). In addition, in dually UBASH3 deficient mouse, TCR stimulation triggers increased tyrosine phosphorylation and ubiquitination of signaling effectors (39). The simultaneous increase of these post-translational modifications could be due to the fact that the activation-deactivation sequence of specific effectors is stopped at a stage where they have been phosphorylated by tyrosine kinases, ubiquitinated by CBL molecules but subsequently improperly dephosphorylated or targeted for degradation as they should when UBASH molecules bind to ubiquitin. As confirmed by recent studies, one of the first targets of this regulation is ZAP-70 (42, 43). In this context, it is possible that CD5 allows molecular cooperativity between CBL and UBASH3 molecules to terminate TCR induced signaling by dampening the activity of ZAP-70 kinase and by contributing to its degradation (Figure 2B).

## CD5-mediated Regulation of CSK

Another potential mediator of the CD5 inhibition that was also detected with both CBL and CBLB after TCR engagement is the tyrosine kinase CSK. The recruitment of CD5 and CSK to both CBL molecules was correlated indicating a possible physical association between them. This association was confirmed by co-immunoprecipation of CSK with CD5 upon TCR stimulation (24). CSK has been shown to control the activity of Src kinases by phosphorylating their C-terminal tyrosine residue (44). In turn, CSK activity depends on its association with the transmembrane adaptor PAG (45, 46). In this context, CD5 ligation was shown to induce the phosphorylation of the Src kinase Fyn at its C-terminal inhibitory residue and attenuate its activity (14). To explain this observation, it has been proposed that CD5 could interfere with the disassembly of the CSK-PAG complexes during T cell activation. However, in contrast to CSK and CD5, PAG was not identified as a binding partner of either CBL or CBLB in TCR stimulated mature T cells. This suggest that different pools of CSK are present in T cells, within different protein complexes. Moreover, a recent study has demonstrated that PAG-regulated TCR signaling is essentially active in effector T cells (47). Thus, it is conceivable that the facilitation of CSK recruitment to the synapse could operate through alternative transmembrane adaptors, and possibly directly with CD5, depending of the activation state of T cells. An attractive model could be that CD5 binds to CSK through its SH2 domain. In this setting, the interaction between CD5 and CSK, induced by TCR stimulation, could participate in a negative feedback loop by reducing the activity of Src kinase recruited to the synapse (Figure 2C).

## CD5 and Immunotherapy

Accumulated knowledge on immunomodulatory properties of CD5 positions this receptor as a putative checkpoint inhibitor, potentially useful in the context of immunotherapies. In this context, one way to harness the inhibitory functions of CD5 would be the development of anti-CD5 monoclonal antibodies (mAb) having diverse functional properties. Thus, mAb with the ability to sequester the receptor away from the T cell synapse could be useful to reduce CD5 inhibitory signaling and increase T cell responses against tumors. Alternatively, anti-CD5 mAbs enhancing the inhibitory role of the receptor could help improve autoimmune diseases by reducing effector functions of autoreactive T cells.

Prior the emergence of antibody-based cancer treatments, results of clinical trials using anti-CD5 mAb have established moderated benefit in patients with chronic lymphocyte leukemia or cutaneous T-cell lymphomas (48, 49). With the recent advances in immunotherapies, experimental protocols have evolved and critical factors have been identified to improve treatment efficacies. For example, manipulation of antibody structure to avoid rapid clearance and immune response against the therapeutic mAb is one of the issue that could be investigated with CD5. Also, evaluation of biological effects provided by combination with other antibodies in a broader spectrum of malignancies could reveal CD5 as a potent target to control cancer.

However, immunotherapy strategies targeting CD5 should be the object of cautious attention. Indeed, as CD5 is expressed in all T cell subsets and on B-1a B cells, *in vivo* administration of CD5 specific antibodies will result in the sum of individual cell type responses. For example, it has been shown that generation of induced Treg (iTreg) cells is altered among CD5 low or CD5 deficient T cell populations (50). It is therefore likely that inhibition of CD5 would simultaneously reduce iTreg cell number and activate effector functions on conventional T cells, thereby increasing T cell reactivity against self and potentiating auto-immune disorders. In addition and as proposed by studies using a mouse expressing a serine-truncated CD5 form, signaling of the receptor can also affect T cell differentiation toward specific Th subsets (51). Therefore, all these parameters must be taken into consideration in order to avoid the onset of undesirable reactions resulting from complex global effects.

## Concluding Remarks

Overall, it appears that distinct molecular mechanisms remain possible to explain the negative regulation of TCR signaling exerted by CD5 in thymocytes, naïve and effector T cells. As illustrated by the different signaling models presented here, CD5 could act as a scaffold coordinating the action of CBL, UBASH3 and CSK molecules within the immunological synapse. In conclusion, CD5 and the identified effectors involved in the same signaling pathway offer great potential for the development of new drugs. However, complexity of the molecular relationships and difficulties to predict perturbations of the system must be taken into account prior to the design of new therapeutic strategies.

## Author Contributions

GV, AGP, and RR generated, analyzed the data and wrote the manuscript.

### Conflict of Interest Statement

The authors declare that the research was conducted in the absence of any commercial or financial relationships that could be construed as a potential conflict of interest.
